# Mid‐life cardiorespiratory fitness and risk of late‐onset dementia incidence

**DOI:** 10.1002/dad2.70239

**Published:** 2026-02-08

**Authors:** Camilla A. Wiklund, Rui Wang, Magnus Lindwall, Sofia Paulsson, Örjan Ekblom, Elin Ekblom‐Bak

**Affiliations:** ^1^ Department of Physical Activity and Health The Swedish School of Sport and Health Sciences Stockholm Sweden; ^2^ Division of Clinical Geriatrics, Department of Neurobiology, Care Sciences and Society Karolinska Institutet Huddinge Sweden; ^3^ Wisconsin Alzheimer's Disease Research Center University of Wisconsin School of Medicine and Public Health Madison Wisconsin USA; ^4^ Department of Psychology University of Gothenburg Gothenburg Sweden; ^5^ Research Department HPI Health Profile Institute Danderyd Sweden; ^6^ Department of Neurobiology Care Sciences and Society Division of Nursing, Health Promotion Among Children and Youth Karolinska Institutet Huddinge Sweden

**Keywords:** cardiorespiratory fitness, dementia, education, late onset, mid‐life, moderation

## Abstract

**INTRODUCTION:**

Cardiorespiratory fitness (CRF) is linked to dementia risk, but moderating factors remain unclear. This study examined how and when CRF in adulthood is associated with late‐onset dementia (> 65 years) and whether sex, civil status, or education moderate this association.

**METHODS:**

In a cohort of 370,980 dementia‐free individuals followed for a mean of 11.9 (standard deviation 6.0) years, CRF was estimated via a submaximal cycle test, with dementia incidence obtained from Swedish National Healthcare Registries.

**RESULTS:**

Results showed that high CRF was associated with lower dementia risk in those under age 55 (hazard ratio [HR]: 0.58, 95% confidence interval [CI] 0.36–0.92) and those > 55 (HR: 0.75, 95% CI 0.63–0.89) at CRF assessment. Medium education levels moderate the association in individuals < 55 years.

**DISCUSSION:**

These findings underscore the role of maintaining a high CRF in dementia prevention, emphasizing education level as a critical moderating factor.

**Highlights:**

- High cardiorespiratory fitness (CRF) is longitudinally associated with a lower risk of late‐onset dementia.
- The association was evident in those both under and over age 55 at CRF assessment.
- Higher education levels moderated the relationship in those < 55 years.
- Sex and civil status did not moderate the association.

## 1 BACKGROUND

Dementia is characterized by a progressive decline in cognitive functions, which severely impairs daily life and social functions.^1^ It is the seventh leading cause of death globally and the leading cause of disability and dependency among older people.^2^ In 2023, > 55 million people worldwide were diagnosed with dementia, and nearly 10 million new cases were estimated each year.^3^

With no effective disease‐modifying treatment available for dementia, attention has shifted toward preventive strategies targeting modifiable factors.^4^ In a recent report by the Lancet Commission, a set of 14 modifiable risk factors throughout the life course was suggested to account for ≈ 45% of risk reduction in dementia.^4^ One of these, regular physical activity, was identified as necessary for sustaining cognitive functions from mid‐life onward, with meta‐analyses and systematic reviews of longitudinal observational studies showing physical inactivity associated with an increased risk of dementia^4^ and Alzheimer's disease (AD).^5^ A factor closely linked to physical activity, cardiorespiratory fitness (CRF), has been suggested as one possible mechanism of how physical activity exerts its effect on dementia risk, as it, apart from genetic contribution,^6^ heavily depends on the current level of moderate‐to‐vigorous intensity physical activity.^7^ In a systematic review and meta‐analysis of prospective cohort studies with CRF measured at one time point in adulthood, low CRF was associated with approximately three times greater risk of dementia later in life compared to high CRF,^8^ with a recommendation to maintain a CRF at 12 metabolic equivalents of task or more (equal to ≥ 42 mL/min/kg) to decrease dementia risk and dementia mortality substantially. In 191 women followed for 44 years, high CRF delayed age at dementia onset by 9.5 years and time to dementia onset by 5 years compared to medium fitness.^9^

**RESEARCH IN CONTEXT**

1. **Systematic Review**: The authors reviewed the relevant literature using PubMed. While the association of cardiorespiratory fitness (CRF) with the overall risk of dementia has previously been studied, there is less knowledge about the moderating effects of factors like education, sex, and civil status influencing the association.
2. **Interpretations**: Our findings showed that CRF was associated with lower risk of late‐onset dementia. The association was present regardless of when CRF was assessed in life, indicating the importance of acquiring and maintaining a healthy CRF in mid‐life. Findings confirmed that education moderates this relationship to benefit those with moderate and high education levels; however, only in those < 55 at the CRF assessment.
3. **Future Directions**: Longitudinal cohort studies with objectively assessed CRF, like this one, are needed to explore this complex relationship further. Detailed information on the differences between types of dementia diagnoses would be highly valuable for making more precise recommendations on prevention strategies.

Several factors are associated with both CRF and dementia risk. For example, with increased age, the risk of dementia, as well as the risk of low CRF, increases.^10^ However, little is known about how CRF may modify the dementia risk during the life span. Understanding when fitness level matters most for dementia risk requires considering potential moderators such as sex,^11^ civil status,^4^, ^12^, ^13^ and education.^14^, ^15^, ^16^ Each is linked to both cognitive health and physical fitness. Sex differences affect physiological responses to exercise and dementia progression, possibly altering the CRF–dementia relationship. Civil status can shape physical activity and dementia risk, influencing how CRF impacts that risk. Education contributes to cognitive reserve and healthier behaviors, which may also modify the association between CRF and dementia. Identifying moderators is essential to address questions about dose–response and identify subgroups with a stronger relation between CRF and dementia.

In this longitudinal cohort study, we will explore how the level of CRF in adulthood is associated with the incidence of late‐onset dementia (> 65 years) later in life in the working population, with special reference to when in life CRF is assessed. Additionally, we aim to study whether any association is moderated by sex, civil status, and educational level.

## 2 METHODS

### 2.1 Population

This is a large‐scale prospective cohort study, including participants who had attended an occupational health service screening (health profile assessment [HPA]) between 1995 and 2022. HPAs have been performed in occupational health services since the early 1970s, and data from the HPAs are stored in a central database. Employees working for a company or organization connected to occupational or other health services in Sweden may be offered to perform an HPA, which is optional and free of charge for the individual. An HPA includes a questionnaire on lifestyle and perceived health questions, a physical examination, and an in‐depth interview with an HPA coach. The HPI (Health Profile Institute; Stockholm, Sweden) is responsible for educating HPA coaches, managing the HPA database, and developing software for data collection. Using the unique Swedish personal identification number,^17^ we followed the individuals in the HPA database over time to see if they developed dementia. They were observed using the Swedish National Patient Registry, the National Cause of Death Registry, and the Swedish Registry for Cognitive/Dementia Disorders (SweDem) until the incidence of dementia, death, or December 31, 2022. We identified 560,399 individuals in the database with valid information on the main variables of sex, civil status, education level, and date of HPA. We excluded individuals without a valid measure of estimated CRF (*n* = 189,125) and information about age (*n* = 7). Further, participants with a diagnosis of dementia before the CRF test were also excluded (*n* = 17). Hence, 370,980 participants were included in the analysis; see Figure S1 in supporting information for more details. All participants gave informed consent to participate in the HPA database at the HPA assessment time point. The Ethical Review Board in Stockholm and the Swedish Ethical Review Authority approved the study (Dnr 2015/1864‐31/2, Dnr 2016 9‐32, Dnr 2019‐05711, Dnr 2022‐03142‐02), and the study adhered to the Declaration of Helsinki.

### 2.2 Exposure

CRF was assessed as estimated maximal oxygen consumption (VO_2_max) using the Åstrand submaximal cycle ergometer test.^18^ The individual cycles for 6 minutes on a constant workload, and the steady‐state heart rate response during the last minute is used to estimate VO_2_max (in L/min, which was further divided by body weight to obtain VO_2_max as mL/min/kg). The Åstrand submaximal protocol has previously been shown to provide valid estimations. A slight non‐significant mean difference at a group level (−0.07 L/min; 95% confidence interval [CI], −0.21 to 0.06 L/min) between V̇O_2_max using the Åstrand test and directly measured V̇O_2_max from maximal effort tests on a treadmill has been shown, with an absolute error and coefficient of variation similar to other submaximal tests (standard error of estimate, 0.48 L/min; coefficient of variation, 18.1%) comparable to directly measured V̇O2max.^19^, ^20^

### 2.3 Outcome

Incident dementia cases were identified by the records in the SveDem, the National Patient Registry, and the National Cause of Death Registry until December 31, 2022. For the latter, dementia was identified as a cause of hospitalization, regardless of whether it was primary, secondary, or tertiary, and so forth, if the person was not identified in SveDem. All dementia cases were identified using the International Classification of Diseases (ICD)‐10 codes (F00‐03 and G30). We excluded early‐onset dementia in our analysis, that is, age of diagnosis < 65 years. Follow‐up time was calculated as the time from HPA until the first dementia diagnosis, death, or the end of the follow‐up period (December 31, 2022).

### 2.4 Covariates

The analyses were adjusted for potential confounders/covariates that could influence the relationship between estimated CRF and dementia risk. We included demographic factors (e.g., age, sex, education, civil status), lifestyle factors (e.g., smoking), health conditions (e.g., multimorbidity), and HPA test date as our covariates. Sex was classified as men or women. Age at HPA was calculated from birth date and HPA date. Education, obtained from Statistics Sweden, was categorized as low (≤ 9 years), medium (12–14 years), or high (> 14 years). Smoking was grouped as never, irregular, or daily. Civil status included married, unmarried, divorced, or widowed. Multimorbidity was defined as the presence of cardiovascular disease (ICD‐10; I00‐I99), cancer (ICD‐10; C00‐D48), psychiatric disorders (ICD‐10; F04‐F09, F20‐F34, F38‐F39), or diabetes (ICD‐10; E08‐E13), each coded 0/1, and summed to yield a score of 0 to 4. All analyses were based on complete cases, and no imputation procedures were applied. Participants with missing data on any covariates included in the models were excluded from the respective analyses.

### 2.5 Statistical analysis

The association between estimated CRF and the risk for late‐onset dementia was analyzed using Cox proportional hazards models, and we reported hazard ratios (HRs) with 95% CIs in all analyses. The proportional hazard assumption was checked using scaled Schoenfeld residuals for all models. The initial intention was to treat age as a continuous variable. However, in the model including all participants, the variable age at HPA testing violated the proportional hazards assumption (*P* < 0.001), and we, therefore, stratified the sample into two groups: > 55 years versus < 55 years when participants attended the HPA test at baseline. The cut‐off threshold was arbitrary and data driven, chosen at 55 years, as this cut‐off provided enough cases in both age groups to analyze the data. The age variable met the proportional hazards assumption within the new strata. We used natural cubic splines to visualize a dose–response relationship for late‐onset dementia, placing knots at the 5th, 50th, and 95th percentiles to balance model flexibility and parsimony and avoid overfitting. The model was adjusted for sex, HPA test date, body mass index (BMI), multimorbidity, education, smoking, and civil status.

Estimated CRF was analyzed as a continuous variable (mL/min/kg) and a categorical variable. The categories were defined as tertiles of estimated CRF within each age group (low, moderate, and high); see Table 1. In the crude model, we analyzed the relationship without adjusting for any covariates. We then adjusted for covariates in the following three steps. In Model 1, we adjusted for sex, HPA test date, and age; in Model 2, we additionally adjusted for BMI; and in Model 3, we additionally adjusted the association for multimorbidity, smoking, education, and civil status.

**TABLE 1:** Baseline characteristics of participants in the total population and after stratification by age into under (<) or over (≥) 55 years.

	Total	< 55 years	≥ 55 years
**Participants, *n* (%)**	370,980	309,097 (83.3)	61,833 (16.7)
**Sex, *n* (%)**			
Female	167,371 (45.1)	137,864 (44.6)	29,507 (47.7)
Male	203,609 (54.9)	171,233 (55.4)	32,376 (52.3)
**Incident dementia, *n* (%)**	1051 (0.4)	197 (0.1)	854 (1.4)
**Mean follow‐up time, years (SD)**	11.9 (6.0)	12.0 (6.1)	11.8 (5.7)
**Mean age at HPA assessment, years (SD)**	42.2 (11.3)	38.8 (9.2)	58.8 (3.0)
**Mean estimated VO_2_max, mL/min/kg (SD)**			
Total continuous variable	36.4 (10.0)	37.7 (10.0)	30.2 (7.4)
Categorical variables			
Low tertile	26.1 (3.7)	27.3 (3.8)	22.5 (2.7)
Medium tertile	35.4 (2.4)	36.7 (2.5)	29.5 (1.8)
High tertile	47.7 (6.7)	49.0 (6.6)	38.5 (4.9)
**Mean BMI, kg/m^2^ (SD)**	25.7 (4.1)	25.6 (4.2)	26.2 (3.8)
**BMI categories, *n* (%)**			
Underweight	8866 (2.4)	7492 (2.4)	1374 (2.2)
Normal weight	174,430 (47.1)	149,922 (48.5)	24,508 (39.7)
Overweight	137,482 (37.1)	110,308 (35.7)	27,174 (44.0)
Obesity	49,835 (13.4)	41,082 (13.3)	8753 (14.2)
**Education, *n* (%)**			
Low (<10 years)	32,571 (8.8)	21,843 (7.1)	10,728 (17.4)
Medium (10–14 years)	173,787 (47.2)	145,660 (47.5)	28,127 (45.6)
High (>14 years)	162,124 (44.0)	139,249 (45.4)	22,875 (37.1)
**Exercise, *n* (%)**			
Often/every day	123,445 (33.6)	103,212 (33.7)	20,233 (33.1)
Sometimes	206,107 (56.1)	171,069 (55.9.7)	35,038 (57.3)
Never	37,604 (10.2)	31,720 (10.4)	5884 (9.6)
**Number of co‐morbidities, *n* (%)**			
No	295,179 (79.5)	258,558 (83.6)	36,621 (59.2)
1	60,350 (16.3)	41,912 (13.6)	18,438 (29.8)
≥2	15,451 (4.2)	8627 (2.8)	6824 (11.0)
**Smoking, *n* (%)**			
Every day	35,165 (9.6)	28,649 (9.4)	6516 (10.7)
Irregular	30,029 (8.2)	27,265 (8.9)	2764 (4.5)
Never	301,839 (82.2)	249,981 (81.7)	51,858 (84.8)
**Civil status, *n* (%)**			
Married	174,022 (47.0)	133,959 (43.4)	40,063 (64.9)
Unmarried	157,883 (42.6)	148,750 (48.2)	9133 (14.8)
Widowed	2705 (0.7)	954 (0.3)	1751 (2.8)
Divorced	35,643 (9.6)	24,813 (8.0)	10,830 (17.5)

Abbreviations: BMI, body mass index; HPA, health profile assessment; SD, standard deviation.

We assessed effect modification by sex, civil status, and education using multiplicative and additive interaction approaches. Multiplicative interactions were tested via product terms in Cox models, with *P*  <  0.05 considered significant. Additive interactions were evaluated using relative excess risk due to interaction (RERI), the attributable proportion (AP), and the synergy index (S) with 95% CIs, and stratified HRs are presented for interpretation. All models were adjusted for the main analysis covariates. Finally, we performed sensitivity analyses excluding those who developed dementia and those who died during the first 5 years after HPA testing to study possible reverse causality. Statistical analyses were performed in 2024 using R, version 4.3.1.^21^

## 3 RESULTS

### 3.1 Participant characteristics

Baseline sociodemographic and lifestyle characteristics of the 370,980 study participants (mean age 42.2 years, standard deviation [SD] 11.3 years) are shown in Table 1. Of the study participants, 6874 (1.9%) were excluded from the analysis in Model 3 due to missing data: education level (2498, 0.7%), smoking status (3947, 1.1%), civil status (727, 0.2%), and BMI (367, 0.1%). During the follow‐up period (mean: 11.9 years, SD 6.6 years), 1051 (0.4%) participants developed dementia. There were 197 (0.1%) cases in the younger age group (< 55 years at HPA assessment) and 854 (1.4%) cases in the older age group (≥ 55 years at HPA assessment). Of those, 56% were classified as having AD, 12% as having vascular dementia, 8% as having dementia secondary to other diseases, and 24% as having other or unspecified dementia. The mean estimated CRF was 37.7 (SD 10.0) mL/kg/min in those < 55 years and 30.2 (SD 7.4) mL/kg/min in those ≥ 55 years.

### 3.2 Association between CRF and incidence of late‐onset dementia

**FIGURE 1:**
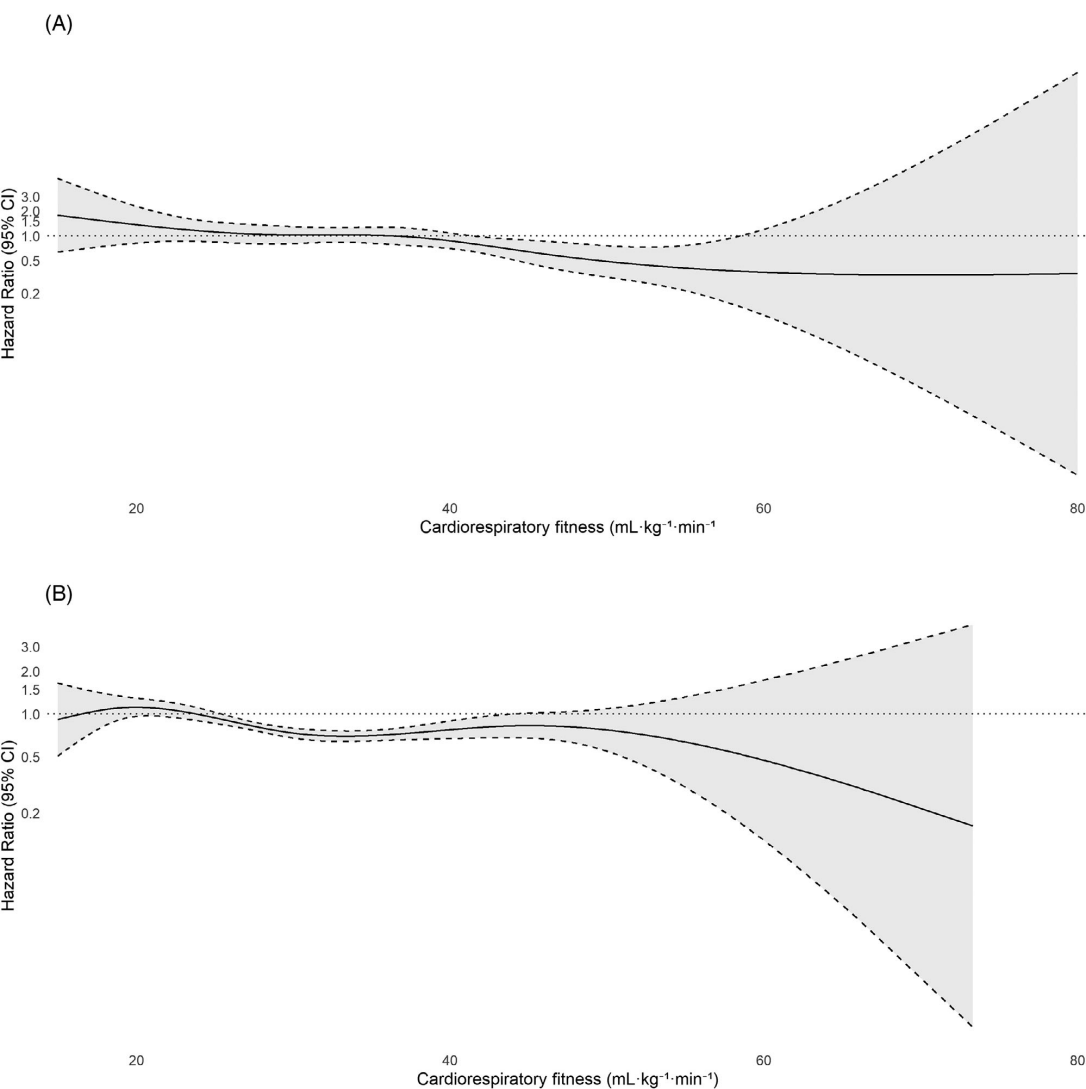
Cox regression with natural cubic splines to model the association of overall dementia incidence and cardiorespiratory fitness (mL/min/kg) in those (A) < 55 years and (B) > 55 years at HPA. Note: Knots set at the 5th, 50th, and 95th percentiles. Cardiorespiratory fitness is included in the model as a continuous measure (mL/min/kg). Models are adjusted for sex, fitness test date, age, body mass index, somatic and psychiatric multimorbidity, smoking, education, and civil status. CRF, cardiorespiratory fitness; HPA, health profile assessment.

The spline regression analysis showed a different pattern of relationships between estimated CRF and dementia risk in those under and above 55 years at CRF testing (Figure 1A,B). In Table 2, the fully adjusted model (Model 3) showed that a one unit increase in estimated CRF (mL/min/kg) was associated with a 3% lower risk of incident dementia among participants with CRF assessments < 55 years of age (Model 3: HR 0.97; 95% CI 0.95–0.99; *P* = 0.008), with a 1% significant association for those > 55 years of age (Model 3: HR 0.99; 95% CI 0.97–1.00; *P* = 0.007). When estimated CRF was treated as a categorical variable (in tertiles), the highest tertile estimated CRF group demonstrated a lower risk of dementia compared to the lowest tertile group (Model 3: HR 0.58; 95% CI 0.36–0.92; *P* = 0.021) in those < 55 years of age. In participants > 55 years, we found a lower risk of dementia in the moderate and high tertile CRF compared to the bottom tertile estimated CRF group (Model 3: HR_moderate_ 0.70; 95% CI 0.59–0.83; *P* < 0.001) and HR_high_ 0.75; 95% CI 0.63–0.89; *P* = 0.001).

**TABLE 2:** Hazard ratio for late‐onset dementia incidence and cardiorespiratory fitness.

		Crude		Model 1^a^		Model 2^b^		Model 3^c^	
	CRF	Estimates (95% CI)	*P* value	Estimates (95% CI)	*P* value	Estimates (95% CI)	*P* value	Estimates (95% CI)	*P* value
**≥ 55 years at HPA (total/cases *N* = 61,883/*n* = 854)**									
	VO_2_max (mL/min/kg)	0.99 (0.98–1.00)	.044	0.98 (0.97–0.99)	0.001	0.98 (0.97–0.99)	<0.001	0.99 (0.97–1.00)	0.007
	Low VO_2_max	1.00 (ref)		1.00 (ref)		1.00 (ref)		1.00 (ref)	
	Moderate VO_2_max	0.75 (0.64–0.89)	<0.001	0.71 (0.60–0.83)	<0.001	0.69 (0.58–0.81)	<0.001	0.70 (0.59–0.83)	<0.001
	High VO_2_max	0.81 (0.69–0.95)	0.011	0.74 (0.63–0.86)	<0.001	0.70 (0.59–0.82)	<0.001	0.75 (0.63–0.89)	0.001
**< 55 years at HPA (total/cases *N* = 309,093/*n* = 197)**									
	VO_2_max (mL/min/kg)	0.94 (0.92–0.95)	<0.001	0.98 (0.96–0.99)	0.004	0.97 (0.95–0.99)	<0.001	0.97 (0.95–0.99)	0.008
	Low VO_2_max	1.00 (ref)		1.00 (ref)		1.00 (ref)		1.00 (ref)	
	Moderate VO_2_max	0.64 (0.47–0.87)	0.005	0.95 (0.70–1.29)	0.736	0.90 (0.66–1.23)	0.518	0.97 (0.70–1.32)	0.833
	High VO_2_max	0.23 (0.15–0.35)	<0.001	0.57 (0.37–0.89)	0.012	0.52 (0.33–0.82)	0.005	0.58 (0.36–0.92)	0.021

Note: CRF is presented as a continuous measure (mL/min/kg) and tertiles of low, moderate, and high. The population is stratified by age at CRF assessment (under and above 55 years).

Abbreviations: BMI, body mass index; CI, confidence interval; CRF, cardiorespiratory fitness; HPA, health profile assessment.

^a^Sex, fitness test date, age.

^b^Sex, fitness test date, age, BMI.

^c^Sex, fitness test date, age, BMI, somatic and psychiatric multimorbidity, smoking, education, civil status.

### 3.3 Moderation by sex, civil status, and education

There were no significant interactions between estimated CRF and sex or estimated CRF and civil status in either age group (Table 3). However, in the younger age group (< 55 years), there was a statistically significant interaction between estimated CRF and education level (*P* = 0.019). This indicates that the relationship between estimated CRF and risk of incident dementia is moderated by education level but not by sex or civil status in individuals < 55 years at HPA testing. The relationship between estimated CRF and education level was further explored in the age‐stratified model in Table 4. The analysis showed a significantly lower risk of incident dementia with higher estimated CRF for medium and high education levels; Model 3: HR_medium_ 0.96; 95% CI 0.95–0.98 (*P* < 0.001) and HR_high_ 0.97; 95% CI 0.96–0.99 (*P* = 0.003) in those < 55 years at HPA testing. On the additive scale, the estimated RERI (0.39, 95% CI: −0.45–1.23), AP (0.32, 95% CI: −0.25–0.89), and synergy index (S = −1.27, 95% CI: −8.05–5.51) suggest a possible positive interaction between high CRF and higher education. However, the wide CIs indicate substantial uncertainty, and there is no statistically significant evidence of additive interaction.

**TABLE 3:** Interaction terms for sex, civil status, and education with CRF stratified by age (over and under 55 years at HPA).

Age group	Interaction term	*P* value
≥ 55 years		
	CRF × sex	
	Women	0.866
	CRF × civil status	
	Married	0.778
	Unmarried	0.916
	Widowed	0.545
	CRF × education	
	Medium	0.132
	High	0.309
< 55 years		
	CRF × sex	
	Women	0.320
	CRF × civil status	
	Married	0.475
	Unmarried	0.204
	Widowed	0.441
	CRF × education	
	Medium	0.019
	High	0.185

Abbreviation: CRF, cardiorespiratory fitness.

**TABLE 4:** Hazard ratio for late‐onset dementia incidence and cardiorespiratory fitness stratified on education level.

		Crude		Model 1^a^		Model 2^b^		Model 3^c^	
	CRF	Estimates (95% CI)	*P* value	Estimates (95% CI)	*P* value	Estimates (95% CI)	*P* value	Estimates (95% CI)	*P* value
**Low education level (total/cases *N* = 32,571/*n* = 253)**									
	VO_2_max (mL/min/kg)	0.95 (0.94–0.97)	<0.001	0.99 (0.97–1.01)	0.274	0.99 (0.97–1.01)	0.265	0.99 (0.97–1.01)	0.444
	Low VO_2_max	1.00 (ref)		1.00 (ref)		1.00 (ref)		1.00 (ref)	
	Moderate VO_2_max	0.68 (0.52–0.90)	0.007	0.87 (0.66–1.16)	0.350	0.87 (0.65–1.16)	0.330	0.90 (0.68–1.21)	0.486
	High VO_2_max	0.42 (0.30–0.57)	<0.001	0.84 (0.59–1.20)	0.342	0.83 (0.58–1.20)	0.329	0.90 (0.62–1.30)	0.564
**Medium education level (total/cases *N* = 171,660/*n* = 469)**									
	VO_2_max (mL/min/kg)	0.92(0.91–0.93)	<0.001	0.97 (0.96–0.98)	<0.001	0.96 (0.95–0.97)	<0.001	0.96 (0.95–0.98)	<0.001
	Low VO_2_max	1.00 (ref)		1.00 (ref)		1.00 (ref)		1.00 (ref)	
	Moderate VO_2_max	0.42 (0.34–0.52)	<0.001	0.67 (0.55–0.83)	<0.001	0.62 (0.50–0.77)	<0.001	0.64 (0.52–0.80)	<0.001
	High VO_2_max	0.18 (0.13–0.23)	<0.001	0.56 (0.42–0.75)	<0.001	0.49 (0.36–0.66)	<0.001	0.51 (0.37–0.69)	<0.001
**High education level (total/cases *N* = 160,307/*n* = 323)**									
	VO_2_max (mL/min/kg)	0.92 (0.90–0.93)	<0.001	0.97 (0.96–0.99)	<0.001	0.97 (0.95–0.99)	<0.001	0.97 (0.96–0.99)	0.003
	Low VO_2_max	1.00 (ref)		1.00 (ref)		1.00 (ref)		1.00 (ref)	
	Moderate VO_2_max	0.50 (0.40–0.64)	<0.001	0.90 (0.70–1.14)	0.378	0.91 (0.71–1.17)	0.462	0.93 (0.72–1.20)	0.582
	High VO_2_max	0.16 (0.11–0.23)	<0.001	0.65 (0.44–0.96)	0.030	0.67 (0.45–1.00)	0.050	0.71 (0.48–1.07)	0.099

*Note*: CRF is presented as a continuous measure (mL/min/kg) and tertiles of low, moderate, and high. The population included in the analysis are those < 55 years at CRF assessment and is stratified on education level (low, medium, and high).

Abbreviations: BMI, body mass index; CI, confidence interval; CRF, cardiorespiratory fitness; HPA, health profile assessment.

^a^Sex, fitness test date, age.

^b^Sex, fitness test date, age, BMI.

^c^Sex, fitness test date, age, BMI, somatic and psychiatric multimorbidity, smoking, education, civil status.

### 3.4 Sensitivity analysis

In the sensitivity analysis performed to address indications of reverse causality, we excluded cases of incident dementia and those who died during the first 5 years of follow‐up (*n* = 25). The results displayed nearly identical effect sizes in the analysis between continuous estimated CRF and later incident dementia in all models. See Table S1 in supporting information for more details.

## 4 DISCUSSION

The findings in the present study show that a higher CRF is associated with a lower incidence of late‐onset dementia in both those < 55 years and those >r 55 years at CRF testing. Furthermore, in those < 55 years at CRF assessment, the highest tertile CRF had a 42% lower incidence of dementia compared to individuals in the lowest tertile. Medium and high education levels moderate the association. In individuals > 55 years of age at CRF assessment, the results showed a slight statistically significant association between continuously measured CRF and risk of late‐onset dementia; in addition, there was a 30% and 25% lower risk for late‐onset dementia comparing those in the moderate and high CRF tertiles to those in the low CRF tertile.

Previous prospective cohort studies have shown a decrease in the risk of dementia with higher CRF levels.^22^, ^23^, ^24^, ^25^ A meta‐analysis found a low CRF to be associated with a nearly three times higher risk of dementia.^8^ Our study confirms these results in this large prospective study population and further extends the previous research by exploring moderating effects. Prior research is not conclusive as to whether the timing or maintenance of physical activity across the lifespan plays a role in later‐life cognition and the risk of dementia. Studies have found that being physically active (compared to no physical activity) and maintaining optimal physical activity are associated with better cognition and a lower risk of dementia at all ages.^26^, ^27^ However, other studies have found no relationship between physical activity and dementia risk and instead suggest that the previously seen relationship can be attributed to reversed causation.^28^ Further, there are also contradictory results found when assessing CRF in individuals with AD. Some research has found individuals with AD to have similar CRF levels, while others have found lower fitness levels compared to healthy controls.^29^ The main results from this study suggest that it is essential to acquire and maintain healthy CRF and physical fitness before age 55 to reduce the risk of dementia. The CRF–dementia association may vary across dementia subtypes because they have different underlying mechanisms. Higher CRF is likely to be more strongly protective against vascular dementia due to its benefits for cerebrovascular health, blood pressure regulation, and prevention of small vessel disease and related ischemic damage.^30^ CRF may also influence AD risk, but more indirectly through effects on inflammation, cerebral perfusion, and neurotrophic processes. Thus, while CRF is generally inversely associated with dementia risk, the mechanisms and strength of this relationship likely differ by subtype and should be considered in future research. There was no moderating effect between CRF and sex or civil status; however, a significant interaction was found between CRF and education. In previous research, education levels have been found to predict the risk of dementia later in life.^14^ A large meta‐analysis found the risk of dementia decreases by 7% for each year increase in education.^16^ Low education level has also been previously shown to predict physical activity in an older population.^15^ This could be part of a possible explanation for why education is moderating the association between CRF and the risk of dementia. The moderating effect of education was strongest before age 55, likely due to mid‐life vascular risk factors and cognitive reserve. Evidence suggests that up to 25% of the education–dementia link is mediated by mid‐life vascular factors.^31^ While low education is associated with lower cognitive reserve, physical activity helps build it.^32^ This may strengthen the cognitive benefits of CRF in mid‐life, when the brain and cardiovascular system are more adaptable. In older age, accumulated vascular and cognitive changes may weaken this effect, suggesting that education helps translate mid‐life CRF into long‐term cognitive resilience. Several potential underlying mechanisms can explain the association between CRF and dementia. Possible mechanisms that have been suggested include improved cerebral blood flow^33^, ^34^ and elevated levels of circulating brain‐derived neurotrophic factor, which enhances neuroplasticity, thereby reducing dementia risk.^35^ Low CRF has also been recognized as a robust and independent predictor of cardiovascular disease and mortality in healthy men and women.^36^ The effect seems to be graded so that even slight variations in CRF are associated with significant risk variations, especially in unfit individuals.^37^, ^38^ Several cardiovascular risk factors (e.g., high blood pressure, triglycerides, and cholesterol) have simultaneously been associated with dementia risk and could potentially act as an underlying mechanism.^39^ However, the higher risk depends on age, as mid‐life high blood pressure seems more strongly associated with a higher risk of dementia than late‐life high blood pressure.^4^, ^39^, ^40^ Considering the relatively young population in this study and the lack of cardiovascular risk factor variables included in the analysis, such conclusions should be made cautiously, and this relationship needs to be further explored in future research.

There are some limitations to consider when interpreting this study's results. First, the study population is relatively young, and therefore, the prevalence of late‐onset dementia cases is low. The data‐driven and somewhat arbitrary age threshold may have influenced the analyses; however, using a higher cut‐off would have greatly reduced the number of younger participants, given the sample's relatively young age, making meaningful analysis impossible. This may lead to low statistical power to detect associations of a smaller magnitude. Age stratification was introduced after detecting a violation of the proportional hazards assumption, improving model fit but limiting the interpretability of age‐specific results. These findings should therefore be considered exploratory. Second, as the data are retrieved from a database of occupational health screenings, the study only includes individuals in the workforce. This limits the generalizability of the results. However, as most of the population in Sweden is part of the workforce, our results still contribute valuable knowledge. Third, CRF was estimated using submaximal testing rather than direct VO_2_max measurement, which was not feasible in this large non‐athletic sample. Although estimation introduces greater measurement error, the submaximal protocol used is considered moderately valid and reliable compared to direct VO_2_max testing. Also, CRF is affected not only by moderate‐to‐vigorous activity but also by genetic factors. We have not been able to take these factors into account in this study. Previous research has found that the association between CRF and the risk of AD is attenuated when accounting for genetic factors.^23^ Residual confounding from lifestyle factors such as diet and alcohol use may remain despite adjustment. These behaviors affect both cardiovascular health and dementia risk and could modestly strengthen or weaken the observed associations. However, any resulting bias is likely small and unlikely to fully account for the findings. Fifth, although excluding dementia cases and deaths in the first 5 years reduced reverse causation, dementia's long preclinical phase means some reverse causality may remain. Last, considering the number of cases in the population, we could not separate cases based on diagnosis. CRF may affect different dementia diagnoses in various ways. The strengths of this study include the large study population, the robust CRF assessment, and the relatively long follow‐up time.

## 5 CONCLUSIONS

In this large, prospective study of the working population in Sweden, results show that higher CRF is associated with a lower risk of late‐onset incident dementia regardless of age at CRF assessment, indicating the importance of acquiring and maintaining healthy CRF before mid‐life. Further, the association was moderated by a higher education level in those ≤ 55 years at assessment. These findings support and extend scientific evidence related to the importance of CRF as a modifiable risk factor for dementia prevention.

## CONFLICT OF INTEREST STATEMENT

Camilla A. Wiklund, Rui Wang, Magnus Lindwall, Örjan Ekblom, and Elin Ekblom‐Bak have no financial or other relationships to disclose. Sofia Paulsson is employed by the HPI Health Profile Institute, which has provided the data used in the study. Author disclosures are available in the supporting information.

## CONSENT STATEMENT

All human subjects provided informed consent to participate in the HPA database at the HPA assessment time point. The Ethical Review Board in Stockholm and the Swedish Ethical Review Authority approved the study.

## Supporting information



Supporting information
